# Dietary Nutrition: The Friend or the Foe to Gastrointestinal Health

**DOI:** 10.3390/nu16234137

**Published:** 2024-11-29

**Authors:** Rosalyn Jurjus, Abdo Jurjus

**Affiliations:** 1 U.S. Food and Drug Administration, Silver Spring, MD 20993, USA; 2 Department of Anatomy, Cell Biology and Physiological Sciences, Faculty of Medicine, American University of Beirut, Beirut 1107, Lebanon; aj00@aub.edu.lb

Over the years and even centuries, instincts, habits, cultures, social determinants, wars, and health needs were, and still are, factors that have shaped our dietary nutrition. Humans are not always capable of obtaining successful dietary nutrition to sustain healthy living and thrive. Globally, one in every nine people is overweight or obese, while about 2 billion people suffer from micronutrient deficiencies [1]. It is also well documented, through multiple studies performed in the past two decades assessing food intake among different dietary patterns, that unhealthy diets are among the top 10 risk factors contributing to the global burden of disease [2,3,4]. Investigations looked into whether it would possible and feasible to come up with innovative and evidence-based customized nutrition protocols to maintain and improve the health status of people. The eight articles in this Special Issue—“Dietary Nutrition and Gastrointestinal Health”—responded to some of these questions, highlighting advances in the field.

Numerous pathologies can alter the physiological mechanisms that guarantee the proper digestion and absorption of nutrients by the gastrointestinal (GI) tract. However, the pathophysiological alterations of such mechanisms, which are necessary for the survival of living organisms, could lead to a wide variety of GI tract disorders [5]. The GI tract is regularly exposed to a wide range of food particles, toxic substances, and potential pathogens that contribute to the development of multiple unhealthy conditions, ranging from cardiovascular diseases to metabolic syndromes, type 2 diabetes mellitus, neurological diseases, Crohn’s disease (CD), ulcerative colitis (UC), colorectal cancer (CRC), celiac disease, irritable bowel syndrome (IBS), gut–brain axis dysregulation, and some cancers, among [6]. The literature is rich in recommended diets and protocols for every disease entity; this Special Issue looks into whether we could reach a stage where we decrease adverse events and improve selective diets for every disorder.

Optimal dietary nutrition and appropriate dietary protocols have a positive impact on the overall health status of the individual by affecting multiple pathways, including the enhancement of anti-inflammatory secretory factors, the preservation of intestinal barrier integrity and permeability, stem cell homeostasis, the regulation of the gut–brain axis, and the maintenance of a healthy GI microbiome, among others [7,8,9,10,11], thus facilitating and promoting the transition to a balanced diet with more diverse nutrient-dense plant foods through consumer education [3]. The modulation of the microbiome and the maintenance of eubiosis is essential, but what is the best strategy to achieve this goal? This Special Issue tackles this important subject and offers new data. The article by Hu and co-workers provides strong evidence in support of the regulatory mechanisms of pectin oligosaccharides (POSs) on cholesterol metabolism through an integral analysis of the gut microbiome using state-of-the-art methodology. Data shows that the fecal gut flora was adjusted positively by citrus POSs, thus increasing “good” bacteria and decreasing the conditional pathogenic ones, therefore enhancing prebiotic activities. They concluded the study by stressing the potential regulatory mechanisms of citrus POSs on cholesterol metabolism through gut microbiome and specific metabolites. 

A wide range of experimental animal studies, as well as human studies, compared various types of dietary protocols by region, such as the Western diet and Mediterranean diet, or plant-based diets compared to meat eaters [3]. Others have explored diets rich in fiber versus fat, as well as their effects on the microbiome and intestinal barrier integrity [12,13]. They demonstrated how a high-fat diet promotes colorectal cancer (CRC) by modulating gut permeability, increasing inflammation, and altering microbiome composition, subsequently leading to dysbiosis and toxic metabolites [12,13,14,15,16,17]. Such points were within the vision and scope of this Special Issue, which provides field data and the appropriate evaluation of some dietary nutrition protocols. The article published by Sayegh and co-authors addressed some of these issues in a case–control study exploring the nutritional profile, disease severity, and quality of life among patients with inflammatory bowel disease (IBD). The study presented original data comparing the dietary habits of patients of various stages of the disease, as well as exploring the relationship of these dietary habits with the quality of life. They compared various related diets and concluded by providing valuable insights into the nutritional profile of a spectrum of Lebanese patients with IBD and how they compare with other populations with distinct nutritional profiles. 

Our research team recently published the highlights of two decades of experience with the gut microbiome in relation to GI diseases like IBD, as well as CRC, based on a large series of experimental animal models and human clinical studies [18,19]. The gut microbiome, known to be a changing ecosystem, contains more than 100,000 billion microorganisms, representing 10–100 times the number of cells in our organism [20,21]; these are mainly bacteria, fungi, viruses, and Archaea. The gut microbiome has been the focus of many studies through the manipulation of its composition via the use of various nutrients, metabolites, and drugs [18]. Consequently, it was recommended that a healthy host–microorganism balance (eubiosis) must be respected to prevent or even treat disease (dysbiosis) and maintain a homeostatic intestinal condition [22]. The essential issue relates to how to modulate the microbiome to reach a homeostatic intestinal flora—is it with prebiotics, probiotics, or other supplements and plant extracts? In this Special Issue, Aureliano Rosa Júnior addressed this important subject using Acanthaceae plant extract. They evaluated the gastroprotective activity of methanolic extracts of this plant, which is traditionally applied in folk medicine in Brazil to treat multiple disorders with clear activity in gastrointestinal disease. The extract is documented to have antioxidant anti-inflammatory activities and is considered to be an estrogenic agent. Data emanating from this animal study using albino mice supported gastroprotective activity and provided a pharmacological basis for its use. 

Multiple experimental research protocols have been published with the expectations of developing interventions that could serve as potential therapeutic targets for the treatment of GI diseases via dietary pulses composed of nutrients and plant extracts [18]. Such protocols have the potential to modulate the gut microbiome’s composition and function, which can promote optimal nutrient digestion and absorption, reduce inflammation, and improve several health conditions such as CVD, immune functions, IBDs, and CRC, among others [15,19,23]. In addition to numerous metabolites, the gut bacteria ferment non-digestible carbohydrates (NDCs) and polyphenols, which results in the production of short-chain fatty acids and phenolic compounds. These metabolites were documented in various animal models to successfully reduce proinflammatory markers and improve inflammation, which is the basic process underlying many pathogenic mechanisms affecting systemic and chronic disorders including those of the GI tract [17,24,25,26]. In this Special Issue, Ocampo Anguiano and collaborators presented original data documenting the benefits of ingesting bean leaves. They reduce metabolic complications, decrease inflammatory markers, inhibit endotoxemia, and improve the lipid profile. The authors concluded that bean leaf consumption improves intestinal integrity. Such a conclusion goes in line with published data from several other studies that are based on specific phytonutrients from foods like the epigallocatechin gallate (EGCG) tea extract, which reduced inflammation in experimental colitis and decreased reactive oxygen species (ROS) expression, in addition to lycopene from tomato or thymoquinone from Nigella Sativa, among others [18].

This Special Issue of *Nutrients* highlighted, through an in-depth review, the controversial role of melatonin as a potential adjuvant therapy in IBD and CRC. Jurjus et al. concluded that melatonin, a neuroendocrine hormone, is involved in the complex interplay between the immune system and gut microbiota, leading to the modulation of gut microbiota in IBD and CRC, and resulting in the provision of protection to the mucosal intestinal barrier with the alleviation of inflammation. Based on the evidence in the literature, the authors suggested a potential use of melatonin as an adjuvant therapy in IBD and CRC. 

This Special Issue also contains a very interesting article by Gallego and co-authors trying to identify a non-invasive biomarker for celiac disease in children. The investigators explored the modulation of zonulin levels in feces and serum. Data showed that as the duration without gluten consumption increased, a significant reduction in fecal zonulin levels was observed. The authors concluded by suggesting the potential use of zonulin, a tight junction protein, as an additional tool to monitor adherence to a gluten-free diet. The importance of this suggested biomarker is that it is non-invasive. 

This Special Issue underlines how an improvement in dietary nutrition, particularly in relation to natural bioactive compounds, can be a real friend to our organisms and can play a fundamental role in the prevention and clinical management of gastrointestinal disorders. It covers, with great relevance, a wide spectrum of both experimental and clinical studies with new emerging data. It contains advances in dietary nutrition categorized either by alimentary tract disease entities and/or by regions or countries related to specific diets with a thorough analysis of their relevance to gastrointestinal health. The studies that feature in this Special Issue foster and widen the spectrum of our knowledge of diet and nutrition in relation to gastrointestinal health, identifying dietary nutrients that are both friend and foe to the gut health.
